# Varus-Valgus Constrained Prostheses in Revision Total Knee Arthroplasty: A Retrospective Study of Mid- to Long-Term Outcomes

**DOI:** 10.7759/cureus.78273

**Published:** 2025-01-31

**Authors:** Kushal Hippalgaonkar, Tarun Jayakumar, Kikkuri Rajeev Reddy, Praharsha Mulpur, Nitish Kohli, A.V. Gurava Reddy

**Affiliations:** 1 Orthopedics, Sunshine Bone and Joint Institute, KIMS-Sunshine Hospitals, Hyderabad, IND

**Keywords:** aseptic loosening, constrained condylar prosthesis, revision total knee arthroplasty, survival analysis, varus-valgus constrained prosthesis

## Abstract

Introduction

Total knee arthroplasty (TKA) is a well-established treatment for end-stage knee arthritis, providing substantial pain relief and functional improvement. However, the increasing need for revision TKA (RTKA) due to factors such as polyethylene wear, aseptic loosening, periprosthetic infection, and instability presents significant challenges. This study aimed to assess the mid- to long-term clinical and radiographic outcomes, implant survival rates, and complications associated with RTKA using a varus-valgus constrained (VVC) prosthesis.

Methods

This retrospective observational study analyzed patients who underwent RTKA with either VVC or condylar constrained knee prostheses at Sunshine Hospitals in Hyderabad, India, between January 1, 2010 and January 1, 2020, with a minimum follow-up of four years. Data were obtained from the joint registry. The inclusion criteria encompassed patients who underwent single-stage or two-stage RTKA for any indication, including periprosthetic joint infection (PJI), instability, or aseptic loosening. Functional outcomes were assessed using the Knee Society Score (KSS) and Oxford Knee Score (OKS), while radiolucent lines were evaluated with the updated Knee Society Roentgenographic Score. Implant survival rates were analyzed using Kaplan-Meier survival analysis.

Results

A total of 139 patients met the eligibility criteria for the final analysis, with a mean follow-up of 6.3 ± 2.4 years. The cohort had a mean age of 64.8 ± 7.8 years, comprising 90 females (64.7%), and a mean BMI of 29.3 ± 5.4. The primary indications for RTKA were PJI (70 cases, 50.4%) and aseptic loosening (47 cases, 33.8%). Significant postoperative improvements were observed in range of motion (from 73.2 degrees preoperatively to 104.3 degrees postoperatively), OKS (from 14.3 to 29.2), and KSS (from 48.2 to 74.9), all of which were highly significant (p < 0.001). Radiolucent lines were detected in 13 femoral components (9.35%) and 18 tibial components (12.95%), with one case progressing to aseptic loosening. Complications included superficial surgical site infections (two cases, 1.4%) and persistent prosthetic joint infections (three cases, 2.2%). Kaplan-Meier survival analysis demonstrated a 98.3% survival rate at 60 months, with reoperation for any cause as the endpoint.

Conclusions

The use of VVC prostheses in RTKA yields favorable long-term outcomes, demonstrating high survival rates and significant functional improvements. While managing bone defects and preventing postoperative infections remain challenges, meticulous surgical techniques and comprehensive postoperative care play a crucial role in achieving reliable results. These factors contribute to improved patient outcomes and enhanced prosthesis longevity in complex RTKA cases.

## Introduction

Total knee arthroplasty (TKA) is widely recognized as the most effective treatment for end-stage knee arthritis, providing significant pain relief and functional improvement [1]. However, with an aging population and a growing number of younger patients undergoing TKA, the demand for revision TKA (RTKA) has increased, posing a significant challenge for surgeons [2]. RTKA, which accounts for approximately 2.4% of all knee arthroplasty procedures, has been on the rise due to factors such as polyethylene wear, aseptic loosening, periprosthetic infection, and instability [2,3].

RTKA is inherently complex, requiring surgeons to address ligamentous deficiency, instability, bone defects, and severe deformities [4]. To enhance implant longevity and minimize the risk of aseptic loosening, strong fixation must be achieved in at least two of the three fixation zones: epiphysis, metaphysis, and diaphysis [5]. In such challenging cases, primary prostheses such as posterior-stabilized (PS) or cruciate-retaining implants are often inadequate, necessitating the use of more constrained designs, such as the semi-constrained varus-valgus constrained (VVC) prosthesis or rotating-hinge knee (RHK) prostheses [6]. While VVC prostheses have demonstrated satisfactory mid-term clinical outcomes in RTKA, their long-term efficacy and survival remain topics of ongoing investigation [7,8].

This study aimed to assess the mid- to long-term clinical and radiographic outcomes, implant survival rates, and complications of RTKA using a VVC prosthesis in the Indian population.

## Materials and methods

This retrospective observational study examined patients who underwent RTKA using either a VVC or condylar constrained knee (CCK) prosthesis. Conducted at Sunshine Hospitals in Hyderabad, India, the study received approval from the Institutional Ethics Committee (SIEC/2022/493) and included RTKAs performed between January 1, 2010 and January 1, 2020. Informed consent was obtained in accordance with ethical guidelines. Data were sourced from the institutional joint registry, encompassing patient demographics, RTKA indications (aseptic loosening, instability, and periprosthetic joint infection (PJI)), surgical details - including prosthesis type, classification of bone defects per the Anderson Orthopaedic Research Institute (AORI) classification - and surgical duration.

The inclusion criteria comprised patients who underwent single-stage or two-stage RTKA for any indication (PJI, instability, or aseptic loosening) with AORI type II or III bone defects [9]. Patients were excluded if they had incomplete preoperative or postoperative functional scores, missing or inadequate clinical and demographic data, unknown implant sizes, or if they declined participation. Additionally, patients who underwent VVC for complex primary TKA or RTKA with PS prostheses or higher levels of constraint (hinge or megaprosthesis) were excluded.

During RTKA, all patients received press-fit tibial and/or femoral porous-coated metaphyseal sleeves. Collected data included age, gender, weight, height, BMI, American Society of Anesthesiologists (ASA) grading, and Charlson Comorbidity Index (CCI). Functional outcomes were assessed using the Knee Society Score (KSS) and Oxford Knee Score (OKS) until the final follow-up. Intraoperative bone loss was classified using the AORI system, where type II defects indicated significant metaphyseal bone loss in the femoral or tibial condyles, while type III defects involved critical metaphyseal segment loss with collateral ligament detachment, often necessitating more constrained revision components.

Surgical technique

All surgeries were performed by a single senior arthroplasty surgeon with over 20 years of experience. A standard medial parapatellar approach was used for exposure in all cases, followed by the removal of synovium and scar tissue from the knee joint. In cases with limited exposure or difficulty in everting the patella, a quadriceps snip was performed. For revisions due to aseptic loosening or instability, primary components were removed using an oscillating saw or osteotomes. In the second-stage procedure for PJI, the antibiotic-laden cement spacer was removed, and re-debridement was carried out.

All patients received the Sigma TC3® Revision Knee System (DePuy, Warsaw, IN, USA) for their RTKA, selected based on ligament status and bone loss at the time of surgery, in accordance with the surgeon’s preference and experience. The femoral and tibial medullary canals were located and reamed until an adequate diaphyseal fit was achieved. The metaphysis was then prepared for a porous-coated sleeve by sequential reaming and broaching to fit the appropriate sleeve size. This was determined based on a snug fit within the metaphysis, without any toggle or play in any direction, and on the restoration of the joint line. After trialing, the final prosthesis was assembled and cemented using Palacos R+G antibiotic cement (Heraeus Medical, Hanau, Germany). The smallest metaphyseal sleeve (size 29) was cemented, while larger sizes were all porous-coated, ensuring cement was not applied to the porous surface of the sleeve.

The standard revision technique was employed to achieve optimal alignment, restore the joint line, balance the flexion-extension gap, ensure proper patellar tracking, and improve range of motion (ROM). Postoperatively, all patients were mobilized according to a standard institutional rehabilitation protocol, with walking and knee ROM exercises starting on the first postoperative day.

Follow-up

Follow-up evaluations were conducted at one month, three months postoperatively, and annually thereafter, with a minimum follow-up duration of four years. Knee function was assessed using the KSS and the patient-reported OKS. Serial radiographic assessments were performed at each follow-up visit to monitor for signs of component failure or issues with bone integration. Radiolucent lines were evaluated using the updated Knee Society Roentgenographic Score [10].

Statistical analysis

The data were analyzed using IBM SPSS Statistics for Windows, Version 24.0 (Released 2016; IBM Corp., Armonk, NY, USA)). Continuous variables were presented as means with SDs, while categorical variables were expressed as frequencies and proportions. Kaplan-Meier survival analysis was used to evaluate survival rates.

## Results

**Figure 1:**
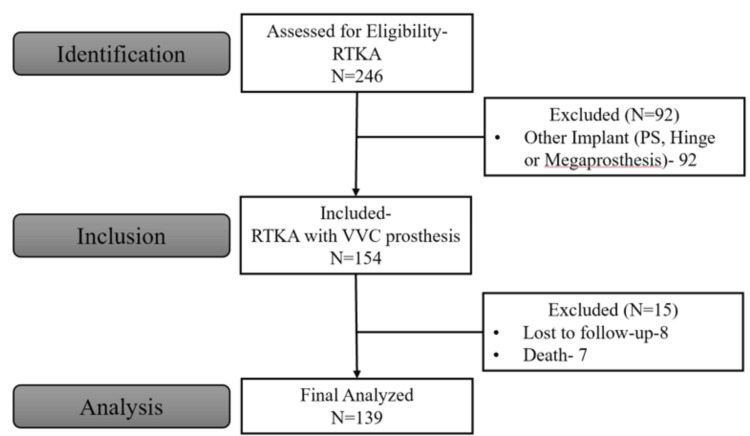
Flowchart of patients included in the study PS, posterior-stabilized; RTKA, revision total knee arthroplasty; VVC, varus-valgus constrained

A total of 139 patients were eligible for final analysis, with a mean follow-up of 6.3 years (SD = 2.4, range: 4-13.3 years) (Figure 1). The mean age of the patient population was 64.8 years (SD = 7.8), with the majority being female (N = 90, 64.7%). The mean BMI was 29.3 (SD = 5.4), and most patients had mild to moderate CCI grading (N = 136, 97.8%). Patient demographics are summarized in Table 1 and Table 2.

**Table 1:** Demographic parameters

Parameter	Mean ± SD
Age (years)	64.84 ± 7.79
BMI (kg/m²)	29.33 ± 5.41
Time since index surgery (years)	6.3 ± 2.4

**Table 2:** Demographic parameters including gender, ASA grade, CCI, and operated side ASA, American Society of Anesthesiologists; CCI, Charlson Comorbidity Index

Parameter	Frequency (proportion)
Gender	
Female	90 (64.7)
Male	49 (35.3)
ASA grade	
1	41 (29.5)
2	88 (63.3)
3	10 (7.2)
CCI grade	
Mild	23 (16.5)
Moderate	113 (81.3)
Severe	3 (2.2)
Side	
Left	74 (53.2)
Right	65 (46.8)

Etiology

The majority of patients had undergone primary TKA for osteoarthritis. The most common indication for RTKA was PJI, accounting for 50.4% (N = 70), followed by aseptic loosening in 33.8% (N = 47). The mean time from primary TKA to revision was 51 months (SD = 34.7), as summarized in Table 3. Nearly half of the patients (N = 68, 49%) underwent single-stage RTKA for various causes.

**Table 3:** Etiology for surgery PJI, periprosthetic joint infection; RTKA, revision total knee arthroplasty; TKA, total knee arthroplasty

Parameter	Frequency (proportion)
Etiology of primary TKA	
Osteoarthritis	98 (70.5%)
Rheumatoid arthritis	21 (15.1%)
Post-traumatic arthritis	20 (14.4%)
Etiology of RTKA	
Infection (PJI)	70 (50.4%)
Aseptic loosening	47 (33.8%)
Instability	21 (15.1%)
Periprosthetic fracture	1 (0.7%)
History of previous surgeries	
Yes	71 (51%)
No	68 (49%)

Manufacturer and implant details

**Figure 2:**
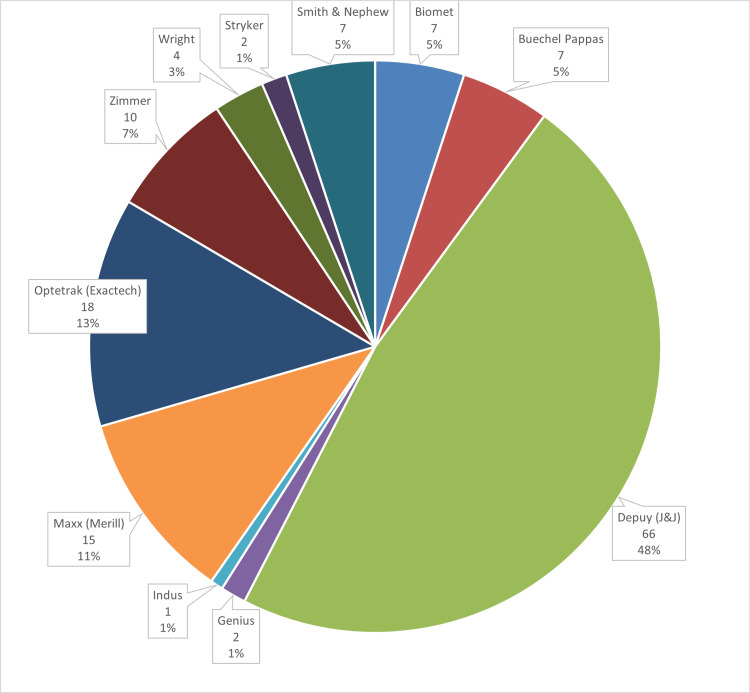
Primary implant manufacturer details

The primary implants used were predominantly the DePuy (J&J) PFC-Sigma (N = 66, 48%), followed by Optetrak (Exactech, Gainesville, FL, USA; N = 18, 13%) and Meril (Maxx) (Meril Life Sciences Pvt. Ltd, Vapi, Gujarat, India; N = 15, 11%), as shown in Figure 2.

All RTKAs were performed using the TC3® implant (DePuy). Cemented femoral and tibial stems were utilized in all cases. Tibial metaphyseal sleeves were used in six cases (4.3%), while femoral augments were employed in 25 knees (18%). Medial tibia wedges were used in nine knees (6.5%). Revision implant details are summarized in Table 4.

**Table 4:** Revision VVC implants used during surgery VVC, varus-valgus constrained

Parameter	Frequency (proportion)
Femur stem length	
75 mm	1 (0.7%)
125 mm	134 (96.4%)
175 mm	4 (2.9%)
Tibia stem length	
30 mm	4 (2.9%)
60 mm	4 (2.9%)
75 mm	99 (71.2%)
115 mm	32 (23%)
Femur augments	25 (18%)
Tibial wedge	9 (6.5%)
Tibial sleeve	6 (4.3%)
Insert thickness (mm)	
10	10 (7.2%)
12.5	20 (14.4%)
15	21 (15.1%)
17.5	41 (29.5%)
20	29 (20.9%)
22.5	18 (12.9%)

Clinical outcomes

The mean ROM demonstrated a highly significant improvement, increasing from 73.2 degrees (SD = 25.2) preoperatively to 104.3 degrees (SD = 9.8) at the final follow-up. Similarly, both the OKS (pre-op: 14.3; post-op: 29.2) and KSS (pre-op: 48.2; post-op: 74.9) showed significant improvements at the final follow-up (p < 0.0001) (Table 5).

**Table 5:** Functional outcomes at preoperative and final follow-up KSS, Knee Society Score; OKS, Oxford Knee Score; ROM, range of motion

Functional score		Mean ± SD	p-value
ROM	Pre-op	73.2 ± 25.2	<0.0001
	Post-op	104.3 ± 9.8	
KSS	Pre-op KSS	48.22 ± 4.25	<0.0001
	Post-op KSS	74.95 ± 14.83	
OKS	Pre-op OKS	14.32 ± 2.61	<0.0001
	Post-op OKS	29.21 ± 6.87	

Radiological outcomes

**Figure 3:**
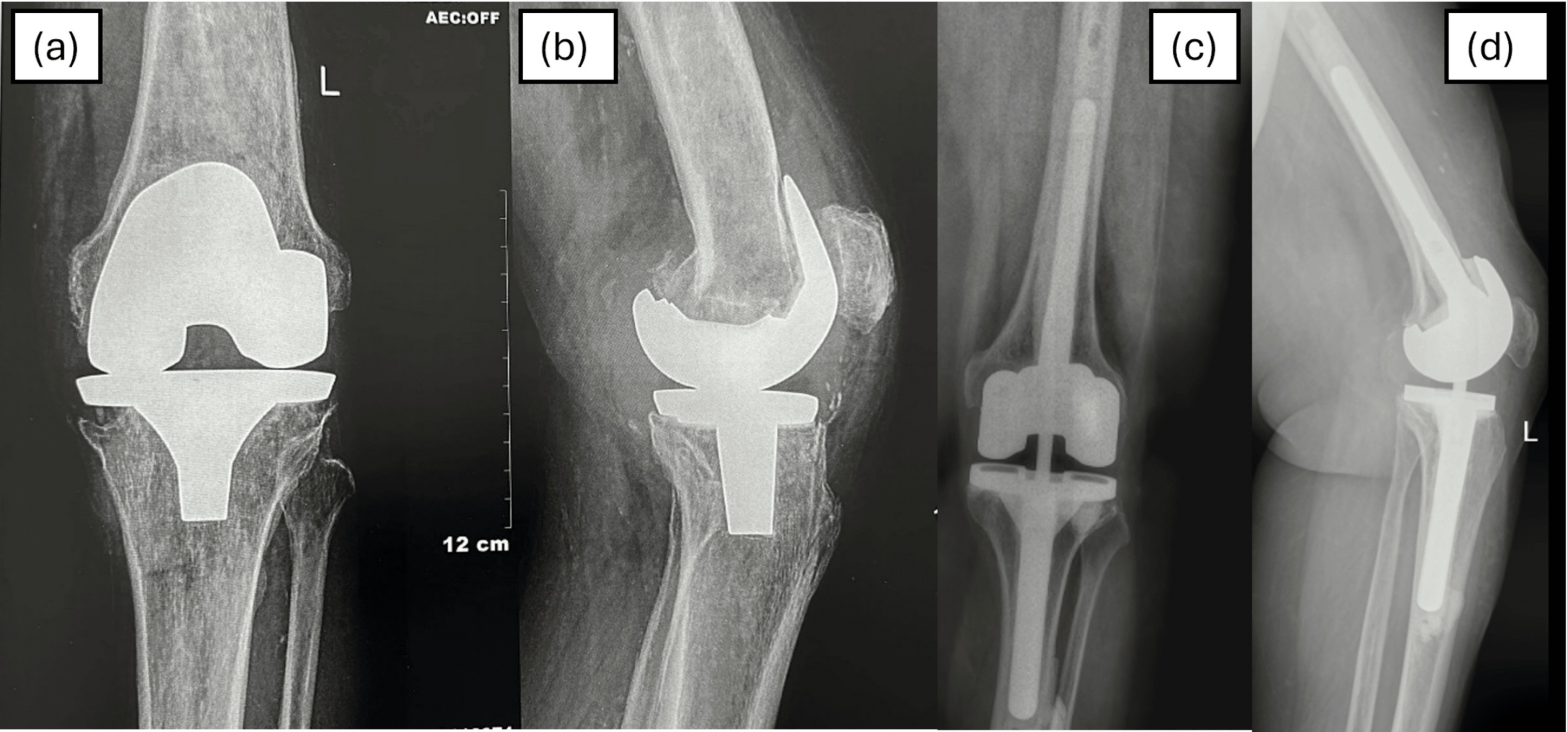
Pre-operative AP (a) and lateral (b) radiographs of the left knee showing aseptic loosening of the femoral and tibial components; AP (c) and lateral (d) radiographs of the same knee after a single-stage revision surgery using the TC-3 prosthesis, showing well-fixed femoral and tibial stems at the final six-year follow-up

Patients demonstrated excellent radiological outcomes (Figure 3). During follow-up, radiolucent lines were observed around the femur in 13 cases (9.35%) and around the tibia in 18 cases (12.95%). However, only one case progressed to aseptic loosening, which was subsequently revised at the 78-month follow-up. No other complications, such as peri-prosthetic fractures, polyethylene insert wear or spin-off, or loosening, were observed.

Complications

Complications occurred in a total of seven patients (5.03%). Two patients (1.4%) developed superficial surgical site infections within three months of revision surgery, which were managed with wound debridement and systemic antibiotics. Three patients experienced persistent PJIs. Among these, two patients ultimately required above-knee amputation after multiple surgeries, with cultures growing *Staphylococcus aureus *and *Pseudomonas* species, respectively. The third patient with persistent PJI underwent knee arthrodesis after a stage 2 revision and multiple debridements and was found to be culture-negative. One patient (0.7%) had aseptic loosening, requiring re-revision at 78 months follow-up for both femoral and tibial components using femoral augments and tibial sleeves. Additionally, one patient (0.7%) experienced postoperative knee stiffness, which was managed with manipulation under anesthesia and aggressive physiotherapy, resulting in satisfactory knee ROM. No other complications, such as symptomatic venous thromboembolism, pulmonary embolism, cerebrovascular accidents, or periprosthetic fractures, were observed (Table 6).

**Table 6:** Complications PJI, periprosthetic joint infection

Complication	Frequency (proportion)	Intervention
Superficial surgical site infection	2 (1.4%)	Wound debridement and systemic antibiotics
Persistent infection (PJI)	3 (2.1%)	Above-knee amputation following multiple debridements, culture-positive *Staphylococcus aureus*
		Above-knee amputation following multiple debridements, culture-positive *Pseudomonas *species
		Knee arthrodesis following multiple debridements, culture-negative
Stiffness	1 (0.7%)	Manipulation under anesthesia
Aseptic loosening	1 (0.7%)	Single-stage revision surgery using TC3 implant with femoral augments and tibial sleeves at 68 months follow-up

Survival analysis

**Figure 4:**
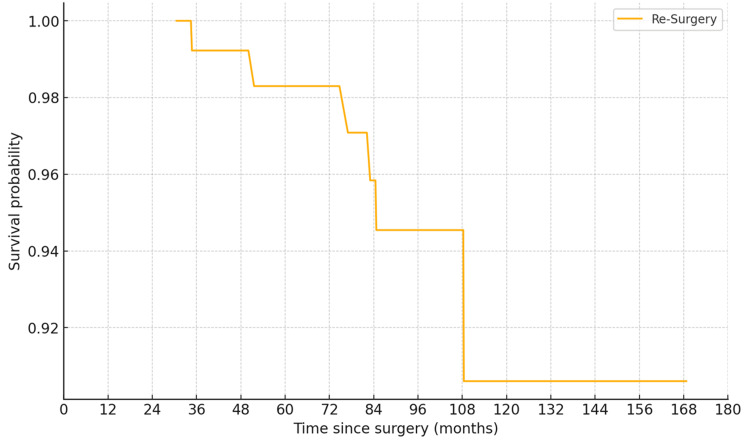
Kaplan-Meier survival graph with reoperation (all-cause) as endpoint

**Figure 5:**
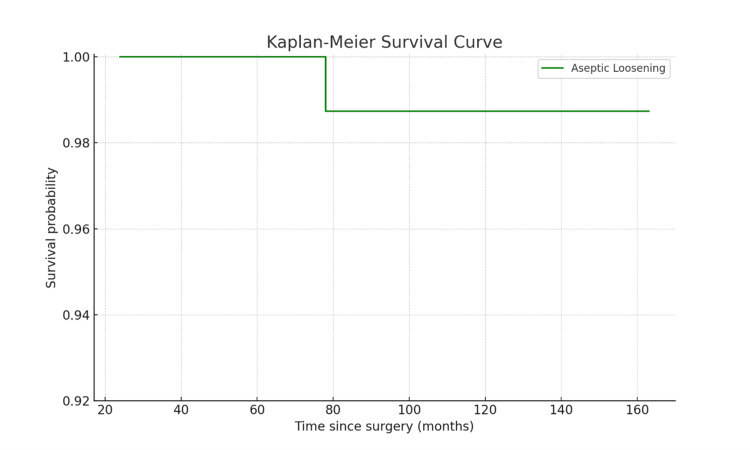
Kaplan-Meier survival graph with aseptic loosening as endpoint

Kaplan-Meier survival analysis, with reoperation for any cause as the endpoint, demonstrated an excellent survival rate. The survival rate at 60 months was 98.3% (95% CI, 95.0-100%) when reoperation for any reason was considered, and 100% (95% CI, 97.5-100%) when death was used as the endpoint (Figure 4). Similarly, the Kaplan-Meier survival curve for aseptic loosening indicated a high survival probability (>98%) for the prosthesis throughout the follow-up period (Figure 5).

## Discussion

The results of this study demonstrate that the use of VVC prostheses in RTKA yields excellent long-term outcomes, with a survival rate of 98.3% at 60 months when reoperation for any cause is considered as the endpoint. This high survival rate is consistent with previously reported results, confirming the effectiveness of VVC prostheses in managing complex knee arthroplasty cases [11-13].

A key finding of our study is the significant improvement in functional outcomes postoperatively. The ROM increased notably from 73.2 degrees preoperatively to 104.3 degrees at the final follow-up. Similarly, both the OKS and the KSS showed substantial improvement, reflecting enhanced patient satisfaction and knee function. These functional benefits underscore the advantages of using VVC prostheses in RTKA, aligning with prior studies that reported similar improvements in knee function and quality of life [14].

However, there remains controversy regarding the use of hinged implants versus VVC implants in RTKA, both in terms of radiological and functional outcomes. Walker et al. questioned the theoretical superiority of VVC implants over RHK implants, showing that RHK implants had lower residual laxity and a better Knee Society Clinical Score [15]. Similar findings were reported by Hossain et al. [16] and Sanz-Ruiz et al. [17]. Nevertheless, two meta-analyses on this topic found no difference in postoperative ROM between the two types of prostheses, with both showing higher postoperative clinical scores in the VVC group [18,19].

Despite these positive outcomes, the study also highlights certain complications associated with RTKA. Specifically, the most common cause of RTKA in our cohort was PJI, followed by aseptic loosening. The rate of PJI in this study was notably high, consistent with findings from other studies that identified infection as a major challenge in RTKA [20]. This emphasizes the need for meticulous surgical techniques and robust perioperative protocols to minimize infection risks.

The management of bone defects is another critical aspect of RTKA. In our study, patients presented with AORI type II or III bone defects, requiring the use of press-fit tibial and/or femoral porous-coated metaphyseal sleeves. The successful integration of these sleeves was reflected in the low rate of aseptic loosening observed. The best method for securing CCK prostheses with stems remains a topic of debate. Daffara et al. found no significant difference in early survival rates between cemented and cementless prostheses in primary TKA [21]. However, in RTKA, cementation of proximal components is widely accepted [22,23]. Hybrid fixation techniques, involving press-fit stems with cement fixation in proximal components, proved reliable, with only one case of aseptic loosening at the last follow-up [24,25].

Interestingly, radiographic outcomes showed nonprogressive radiolucent lines in 13 femoral and 18 tibial components, with only one case progressing to aseptic loosening at 78 months postoperatively. This suggests that while radiolucent lines are relatively common, they do not necessarily predict clinical failure if they remain nonprogressive. This aligns with previous studies indicating that nonprogressive radiolucent lines are not always associated with poor clinical outcomes [26].

The complication rate in our study was 5.03%, with two cases of superficial surgical site infection and three cases of persistent PJI. Management included wound debridement, systemic antibiotics, and, in severe cases, above-knee amputation or knee arthrodesis. The incidence of these complications highlights the complex nature of RTKA and the importance of comprehensive postoperative care and monitoring [27]. The Kaplan-Meier survival analysis confirmed the robustness of our findings, with a survival rate of 98.3% (95% CI, 95.0-100%) at 60 months with reoperation for any cause as the endpoint. These results are comparable to those reported in other studies evaluating the long-term survival of CCK prostheses in RTKA [7,12,13].

This study has limitations, including its retrospective design and reliance on data from a single high-volume tertiary care institute with a highly experienced surgeon, which may limit the generalizability of the findings [28]. Additionally, while the follow-up period was sufficient to demonstrate long-term outcomes, late failures or complications may arise beyond the observed time frame, which will be addressed in future studies. Furthermore, the lack of power analysis, subgroup analysis (based on age, BMI, and type of complication), and a comparative implant cohort are limitations of this study. Future research should aim to include larger patient cohorts with longer follow-up periods, potentially incorporating multicenter data to enhance the robustness and applicability of the findings.

## Conclusions

The use of VVC prostheses in RTKA shows promising mid- to long-term outcomes, characterized by high survival rates and substantial improvements in functional scores. While challenges such as managing complex bone defects and minimizing postoperative infections persist, this study underscores the importance of meticulous surgical techniques and comprehensive postoperative care in achieving favorable results. Future prospective studies with larger cohorts and multicenter data are essential to validate these findings, offering valuable insights for optimizing prosthetic designs and refining surgical techniques.
